# Successful treatment of extensive spinal epidural abscess with fluoroscopy-guided percutaneous drainage: a case report

**DOI:** 10.1186/s40981-020-0309-z

**Published:** 2020-01-15

**Authors:** Masashi Fujii, Tsutomu Shirakawa, Nobuaki Shime, Yasuyo Kawabata

**Affiliations:** 1 Department of Anaesthesia, Nagahama Red Cross Hospital, 14-7 Miyamae-cho, Nagahama, Shiga 526-8585 Japan; 2 Department of Orthopedic Surgery, Nagahama Red Cross Hospital, Nagahama, Shiga Japan; 3 Department of Emergency and Critical Care Medicine, Graduate School of Biomedical & Health Sciences, Hiroshima University, Hiroshima, Japan

**Keywords:** Spinal epidural abscess, Percutaneous drainage, Fluoroscopy

## Abstract

**Background:**

Surgical drainage and antimicrobial therapy are the most accepted empirical treatments for spinal epidural abscess. However, surgery may not be indicated when patient’s general health condition is poor. Percutaneous drainage has been reported as a non-surgical treatment for children or patients with no or minor neurological deficits. Here we describe the successful treatment of an extensive spinal epidural abscess with fluoroscopy-guided percutaneous drainage in an elderly man with progressive muscle weakness who could not be operated because of a poor general health condition.

**Case presentation:**

An 81-year-old man presented with fever, back pain, and progressive muscle weakness in bilateral legs. Magnetic resonance imaging (MRI) showed extensive fluid retention in the spinal epidural space (Th6 to L3). Paraplegia due to an epidural abscess was suspected. We considered an emergency operation; however, the patient’s general condition was poor. Therefore, fluoroscopy-guided percutaneous epidural drainage was performed. After drainage, his back pain and muscle weakness gradually resolved. After 3 weeks, MRI showed that the abscesses had completely disappeared.

**Discussion:**

Compared with surgical drainage, fluoroscopy-guided percutaneous epidural drainage is a less invasive treatment option for patients with a poor general condition.

## Background

The mortality rate for epidural abscess is high [1]; therefore, early diagnosis and treatment are essential. Evidence shows that surgical drainage and antimicrobial treatment are preferred [2–5]. However, surgery is not considered when a patient’s general health condition is poor. Percutaneous puncture drainage has shown occasional success in previous reports, although the indications are unclear [6, 7]. Here we describe the successful treatment of an extensive spinal epidural abscess with percutaneous epidural puncture drainage in an elderly patient with progressive muscle weakness who could not be operated because of a poor general health condition.

## Case presentation

Written informed consent was obtained from the patient and his family for publication of this case report and accompanying images.

An 81-year-old man (height, 175 cm; weight, 85 kg) visited the emergency department with bilateral leg weakness, which led to difficulty in knee flexion 3 days before. He also developed fever and experienced back pain for approximately 1 week. Earlier, he had been walking with the assistance of a walker. He reported a history of aortic valve replacement surgery, mitral valvuloplasty, and tricuspid valvuloplasty, in addition to a history of heart failure, atrial fibrillation, obstructive arteriosclerosis, diabetes, chronic obstructive pulmonary disease, spinal canal stenosis, and dementia. Therefore, his general health condition was deemed poor. He was taking aspirin, apixaban, furosemide, spironolactone, and insulin. Echocardiography showed poor wall motion with an ejection fraction of 41% and pulmonary hypertension (65/13 mmHg).

We were unable to obtain detailed information regarding neurological symptoms such as sensory disturbance and movement disorder because of the severe dementia. He could not flex his right knee, although he could briefly flex his left knee in the supine position. He also complained of severe back pain (face rating scale [FRS] = 4) at rest, which increased with body movement (FRS = 5). Except for an elevated temperature (38.0 °C), his vital signs were stable. Blood examination revealed a slight increase in C-reactive protein (3.64 mg/dl) and white blood cell count (10,300/μl).

**Fig. 1:**
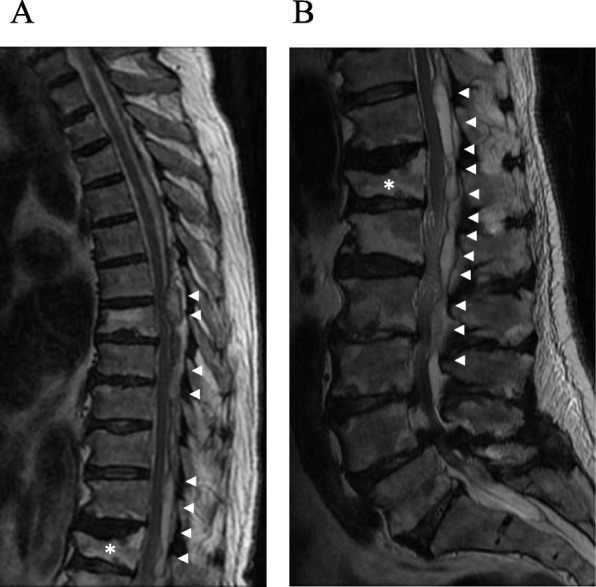
**a**, **b** Magnetic resonance imaging (T2) performed on admission. Fluid retention is observed in the epidural space behind the Th6-L3 spinal canal (arrow). Compression of the spinal cord near Th6/7, Th11/12, and L2/3 due to fluid retention is shown. Vertebral compression fractures can also be seen at Th12 (asterisk)

**Fig. 2:**
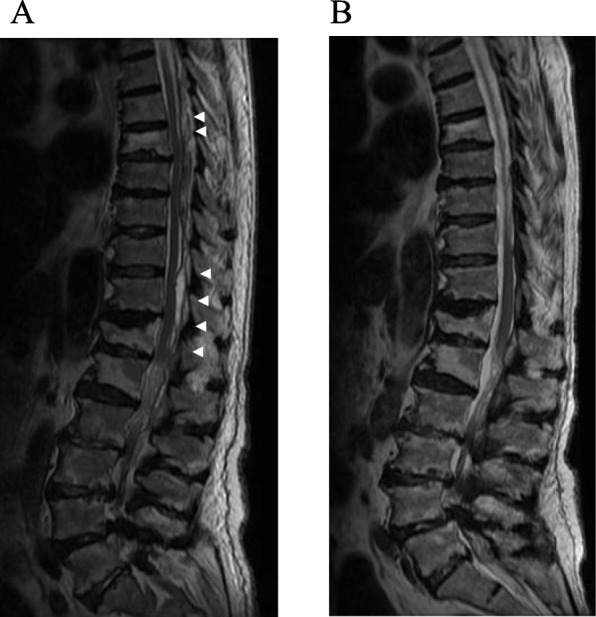
**a** Magnetic resonance imaging (MRI; T2) performed a day after fluoroscopy-guided percutaneous puncture. The volume of fluid in the epidural space has decreased and the spinal cord compression has been relieved (arrow). **b** MRI (T2) performed after 3 weeks. The epidural fluid pool has completely disappeared

Magnetic resonance imaging (MRI) showed extensive fluid retention in the spinal epidural space (Th6 to L3) and compression of the spinal cord (Th6/7, Th11/12, L2/3) (Fig. 1). The latter suggested paraplegia due to an epidural abscess. We performed emergency drainage using fluoroscopy-guided percutaneous puncture. The patient was placed in the prone position and received local anesthesia, following which any abscess near Th6/7, Th11/12, and L2/3 was punctured using a 14 G Tuohy needle under fluoroscopic guidance. Approximately 3 ml of purulent discharge was drained from the Th6/7 and L2/3 levels, and approximately 4 ml of a clear, light-yellow colored discharge was drained from the Th11/12 level. For involved areas near Th6/7, two needles were placed at the Th6/7 and Th8/9 levels, respectively. A 5-Fr catheter was placed under fluoroscopic guidance using a guide wire, followed by perfusion with saline until apparent removal of the corpuscles. MRI performed 1 day later showed relief from the compression due to the abscess. Three weeks later, the abscess had completely disappeared (Fig. 2).

At the time of admission, provisional treatment with meropenem, clindamycin, and vancomycin was initiated and continued for 3 days. The prescription was de-escalated to cefazolin and clindamycin after group G streptococcus was detected in the abscess, and the treatment was continued for 28 days. Blood cultures were negative. Pain on movement (FRS = 2) reduced after the drainage procedure, while pain at rest also improved (FRS = 1) after 3 days. The muscle weakness gradually resolved, and he could walk to a portable toilet without assistance 20 days after the procedure. He was discharged to a nursing home for the elderly on the 57th day.

## Discussion

The mortality rate for spinal epidural abscess is high, reaching 16% [1]. Preoperative neurological findings are important predictors of the final neurological prognosis [2]. However, paralysis may progress rapidly, and immediate intervention is required after diagnosis [8–10]. Surgical drainage and antimicrobial therapy are the most accepted empirical treatments [2–5]. Although there are several criteria for surgical treatment [3, 11], contraindications are also reported [12, 13]. Non-surgical treatment is an alternative; however, antibiotic therapy alone is indicated only for select patients [14].

In the present case, surgical indications were determined through Baker’s criteria. The abscess was extensive and involved multiple levels, and extensive decompression was necessary. However, the patient would not be able to tolerate prolonged surgery in the prone position because of poor cardiac function. Furthermore, there were concerns about massive bleeding during and after surgery because he was taking aspirin and apixaban. The incidence of epidural hematoma after spinal surgery is 0.1 to 3% [15, 16], and extensive surgery and preoperative coagulopathy are risk factors [17]. Moreover, the risk of surgical site infection was high because of the severe diabetes. Because more than 3 days had passed since the onset of muscle weakness in his legs, we decided not to perform surgery.

To the best of our knowledge, there are no reports of percutaneous drainage in a patient with a poor general condition deemed unsuitable for surgery. Percutaneous drainage can be considered for children [18, 19], patients with no or minor neurological deficits, or patients with an abscess that is resistant to antibiotic therapy [20]. Our results have suggested that even in patients with severe neurological symptoms, drainage could facilitate neurological recovery due to immediate spinal decompression.

Increased risk of spinal epidural hematoma was also anticipated due to anticoagulant. However, the frequency of spinal epidural hematoma after epidural anesthesia in patients taking the anticoagulant heparin is 1/3,100, which is lower than that after surgery [21, 22].

In conclusion, fluoroscopy-guided percutaneous epidural drainage is a less invasive treatment option for patients with a spinal epidural abscess and a poor general health condition.

## Data Availability

Data sharing is not applicable to this article as no datasets were generated or analyzed during the current study.

## Abbreviations

FRS: Face rating scale
L: Lumbar
MRI: Magnetic resonance imaging
Th: Thoracic
